# Simultaneous Determination and Stability Studies on Diminazene Diaceturate and Phenazone Using Developed Derivative Spectrophotometric Method

**DOI:** 10.1155/2017/4269587

**Published:** 2017-01-26

**Authors:** Ruaa Mohamed Akode, Shaza Wagiealla Shantier, Elrasheed Ahmed Gadkariem, Magdi Awadalla Mohamed

**Affiliations:** Department of Pharmaceutical Chemistry, Faculty of Pharmacy, University of Khartoum, P.O. Box 1996, Khartoum, Sudan

## Abstract

This work presents UV first derivative spectrophotometry as a precise, accurate, and feasible method for simultaneous determination of diminazene diaceturate and phenazone in bulk and dosage forms. The absorbance values of diminazene diaceturate and phenazone aqueous mixture were obtained at 398 nm and 273 nm, respectively. The developed method was proved to be linear over the concentration ranges (2–10) *μ*g/mL and (2.496–12.48) *μ*g/mL for diminazene diaceturate and phenazone, respectively, with good correlation coefficients (not less than 0.997). The detection and quantitation limits were found to be (LOD = 0.63 and 0.48 *μ*g/mL; LOQ = 1.92 and 1.47 *μ*g/mL, resp.). The developed method was employed for stability studies of both drugs under different stress conditions. Diminazene diaceturate was prone to degrade at acidic pH via first-order kinetics. The degradation process was found to be temperature dependent with an activation energy of 7.48 kcal/mole. Photo-stability was also investigated for this drug.

## 1. Introduction 

**Figure 1:**
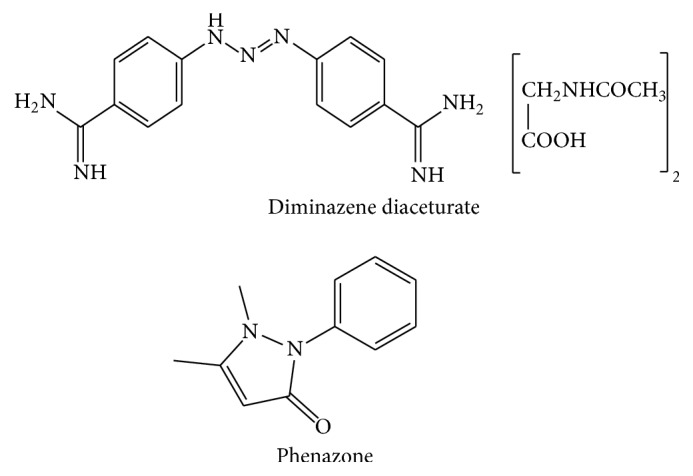
Chemical structure of diminazene diaceturate and phenazone.

The combination therapy of diminazene diaceturate (DD) and phenazone (PHZ) (Figure 1) is prescribed in veterinary field for the treatment of trypanosomiasis and babesiosis. The trypanocide and babesiacide effects mainly return to DD [1]. It is an aromatic diamidine derivative with acidic properties [1]. PHZ is a pyrazolone derivative with basic properties used as anti-inflammatory and stabilizer for DD aqueous preparation since the latter is only stable for 2-3 days [2, 3].

Despite their wide use in developing countries, DD and PHZ formulations have no official method for their routine analysis and stability evaluation. Nevertheless, literature review revealed various methods for their analysis. The majority of the reported methods were concerned with the analysis of these drugs separately in biological fluids or tissues rather than pharmaceutical dosage forms. These methods include different chromatographic, ELISA, and radioassay techniques [4–12]. RP-HPLC and absorption and ratio spectra wavelet approaches were reported for the simultaneous determination of DD and PHZ in pharmaceutical combinations [3, 13–15]. The stability profile of DD and PHZ was also assessed using various chromatographic methods [3, 13, 16, 17]. However, most of these methods are tedious and expensive for routine analysis. Besides its simplicity, spectrophotometry and particularly derivatization of normal spectrum (zero order) was shown to have many advantages such as resolution of overlapping peaks and background elimination [18]. These properties make derivatization of spectrum an alternative stability indicating method instead of HPLC.

Therefore, the aim of this work was to develop a feasible, accurate, and precise first derivative spectrophotometric method for the simultaneous determination and stability studies of DD and PHZ in bulk and pharmaceutical dosage forms.

## 2. Materials and Methods

### 2.1. Instrumentation

All spectrophotometric measurements were conducted on Shimadzu UV 1800 spectrometer (ENG 240 V, Japan) using quartz cells (1 cm), scanning range 200–450 nm. Heating was conducted on temperature regulated water bath (YCM-010E, Germany).

### 2.2. Drugs

Sachet of commercial brands (2.56 g) consisting of DD (1.05 g) and PHZ (1.31 g) was purchased from local market. Both DD (100.3%) and PHZ (99.9%) reference standards were kindly provided by National Drug Quality Control Laboratory.

### 2.3. Reagents

NaOH, HCl (10 M), and H_2_O_2_ (3%) were of analytical grade (BDH Chemicals Ltd. Poole, UK). McIlvaine buffer (pH 2.2) was prepared by mixing citric acid and disodium hydrogen phosphate solutions [19].

### 2.4. Stock Solutions of Standard and Sample

Standard stock solution consisting of a mixture of DD and PHZ was freshly prepared in distilled water to give a final concentration of 50 *μ*g/mL (DD) and 62.4 *μ*g/mL (PHZ). Samples stock solutions were prepared in similar manner to give the same concentration.

### 2.5. Method Validation

#### 2.5.1. Linearity

Different aliquots of the standard stock solution were diluted to obtain final concentration range of 2–10 *μ*g/mL (DD) and 2.496–12.48 *μ*g/mL (PHZ). The first derivative spectrum was recorded and the absorbance values were plotted against concentrations to obtain the calibration curves. The linearity data was then interpreted from calibration curves derived from five different analytes concentration.

Aliquots of sample stock solution were treated as under linearity. The sample content was calculated by direct comparison of sample/standard absorbance values.

#### 2.5.2. Limits of Detection (LOD) and Quantitation (LOQ)

Limits of detection and quantitation were interpreted according to the following equations:

(1) LOD=3.3δS;LOQ=10δS,

where *δ* is the standard deviation of the intercept of the regression line and *S* is the slope of the calibration line [20].

#### 2.5.3. Precision and Accuracy

To evaluate method precision, different concentrations were prepared from sample stock solution and analysed three times within the same day and in three separate days to evaluate within-day and between-day precision, respectively. Recovery studies were conducted to assess the accuracy through spiking of sample (8 *μ*g/mL (DD); 9.98 *μ*g/mL (PHZ)) with standard of 50%, 100%, and 150% of the sample concentration (*n* = 3). The following equation was used to calculate % recovery:

(2) $$\begin{array}{c} \%\text{ recovery}=\frac{\left(A_{mix}-A_{sam}\right)}{A_{std}}\times 100, \end{array}$$

where *A*_mix_ is the absorbance of the mixture solution, *A*_sam_ is the absorbance of the sample solution, and *A*_std_ is the absorbance of the standard solution [20].

### 2.6. Stability Indicating Studies

#### 2.6.1. Effect of Acidic, Alkaline, and Oxidative Conditions on Stability of DD Standard Solution

3 mL of DD standard solution (20 *μ*g/mL) was mixed with 2 mL of either 0.05 M HCl or 0.1–1 M NaOH. The volume was completed to 10 mL with distilled water. The first derivative spectra were then recorded at suitable time intervals (10 minutes). The effect of oxidative conditions on DD standard solution was investigated by repeating the same procedure using 1 mL of 1.2 M H_2_O_2_ instead of NaOH or/and HCL. The responses were recorded every day for three days using the developed method.

#### 2.6.2. Effect of Light on Stability of DD Standard Solution

DD standard solution (6 *μ*g/mL) was placed in transparent and amber glass tubes. The solutions were then subjected to direct sunlight during mid-day time for 4 hours. Another DD standard solution (6 *μ*g/mL) was placed in transparent glass tube and irradiated with UV light (254 nm) for 24 hours. The degradation process was monitored using the developed method.

#### 2.6.3. Temperature Effect on Stability of DD Standard Solution

Aliquots of DD standard solution (3 mL, 20 *μ*g/mL) were transferred into four stoppered glass tubes. 1 mL of McIlvaine buffer solution (pH 2.2) was added to each tube. The solutions were heated at 40°C at suitable time interval (5 minutes). After cooling, the volumes were completed to 10 mL with distilled water and the first derivative spectra were recorded. The same procedure was repeated at heating temperature 50°C.

#### 2.6.4. Effect of Acidic, Alkaline, and Oxidative Conditions on Stability of PHZ Standard Solution

5 mL of PHZ standard solution (24.8 *μ*g/mL) was placed in 25 volumetric flask. 2 mL of either 1 M HCL or 0.1 M NaOH was added and the volume was completed to the mark with distilled water. The solutions were placed at room temperature and heated at 100°C. The first derivative spectra were recorded every 15 minutes. The effect of oxidative conditions on PHZ was investigated by adding 1 mL of 1.2 M H_2_O_2_ to 3 mL PHZ standard solution (24.8 *μ*g/mL). The volume was completed to 10 mL with distilled water and first derivative spectra were recorded every day for three days.

#### 2.6.5. Effect of Light on PHZ Standard Solution

PHZ standard solution (7.49 *μ*g/mL) was placed in transparent and amber glass tubes. The solutions were then subjected to direct sunlight during mid-day time for 4 hours. The degradation process was monitored using the developed method.

## 3. Results and Discussion

### 3.1. Method Development

**Figure 2:**
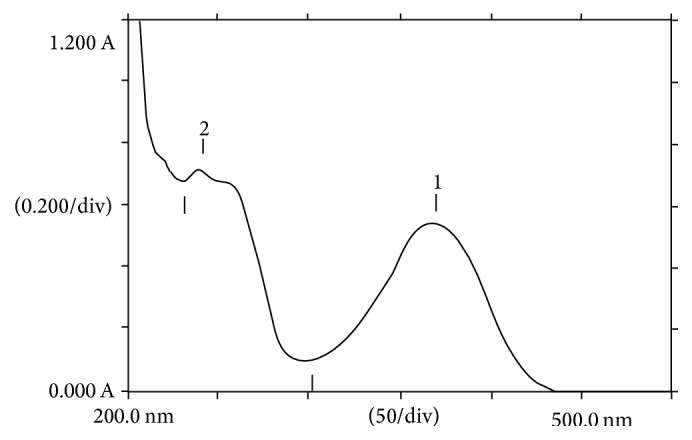
Normal spectrum of DD (1) and PHZ (2) aqueous mixture.

**Figure 3:**
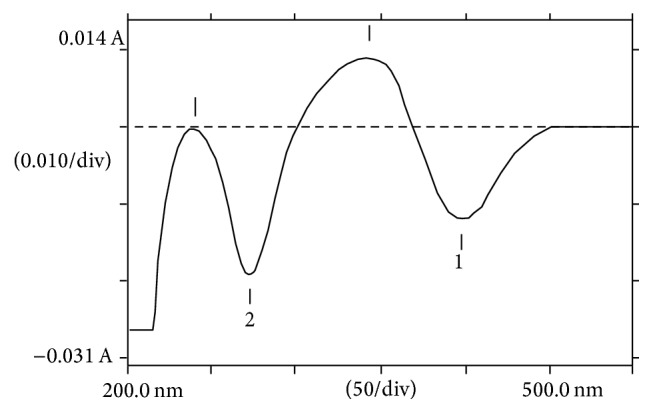
First derivative spectrum of DD (1) and PHZ (2) aqueous mixture.

The normal spectrum of DD and PHZ mixture revealed *λ*_max_ at 370 nm and 241 nm, respectively (Figure 2). Since derivatization of normal spectrum gives rise to reduction of band width, sharper peaks were achieved on first derivative spectrum and DD and PHZ were successfully resolved at 398 nm and 273 nm, respectively (Figure 3).

### 3.2. Validation of the Developed Method

The proposed method was validated in accordance with ICH guidelines by assessment of different parameters [21].

#### 3.2.1. Linearity, LOD, and LOQ

The developed method was used to construct DD and PHZ standard calibration curves. The linearity data was interpreted at 95% confidence limit and summarized in Table 1.

According to ICH, linearity parameters that should be estimated are slope, intercept, correlation coefficient, and residual sum of squares. As tabulated above, the correlation coefficient was closer to unity (>0.997). Further, the 95% confidence interval of the intercept included the theoretical zero which in contrast was not included within slope confidence interval. It was also found that residual sum of squares was closer to zero with residual values that were randomly distributed between negative and positive values. LOD and LOQ values are smaller than a reported chromatographic method [14]. These findings greatly confirmed the linearity of the regression model of first derivative spectrophotometry.

The content uniformity of sample was analysed using the developed method where good results were obtained (100.12%  ±  1.45%; 100.11%  ±  0.82%; *n* = 3) for DD and PHZ, respectively.

#### 3.2.2. Precision and Accuracy

Precision studies revealed RSD% values within acceptable limits (<3%) reflecting the precision and robustness of the method within the selected range (Table 2). The results of “% recovery” were between 88.32% and 90.96% for DD and between 96.84% and 101.12% for PHZ which in turn reflected the accuracy of the method.

### 3.3. Stability Studies on DD

**Figure 4:**
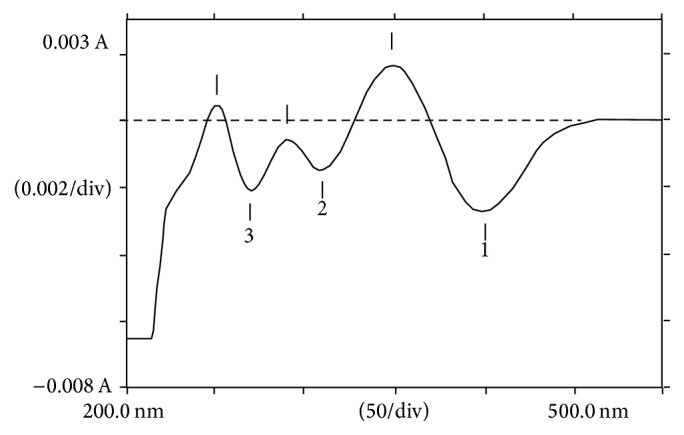
First derivative spectrum of DD (1) and its degradation products (2, 3) in acidic conditions.

**Figure 5:**
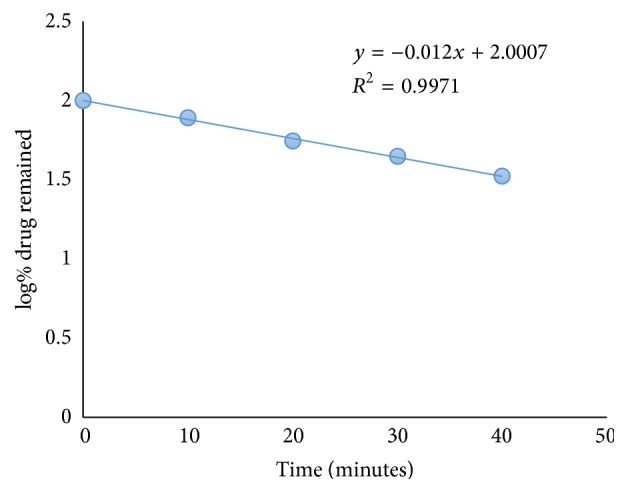
Degradation of DD in acidic conditions (0.05 M).

Forced degradation conditions were employed to investigate stability of DD, stability indicating power, and specificity of the developed method. The effect of different acid concentrations at different time intervals on DD degradation rate was investigated using the developed method. The first derivative spectrum of DD solution treated with 0.05 M HCL reflected a decrease in its peak at 398 nm with appearance of well resolved degradation products peaks at 313 nm and 271 nm (Figure 4). The presence of triazene bridge in DD structure allows it to be susceptible for degradation. These degradation products were already confirmed to be 4-aminobenzamidine and 4-hydroxybenzamidine [16]. The degradation rate was calculated from the plot of log% remained drug versus time interval (Figure 5) and found to follow first-order kinetics. Values of *t*_1/2_ and *t*_90_ were found to decrease with increased time.

**Figure 6:**
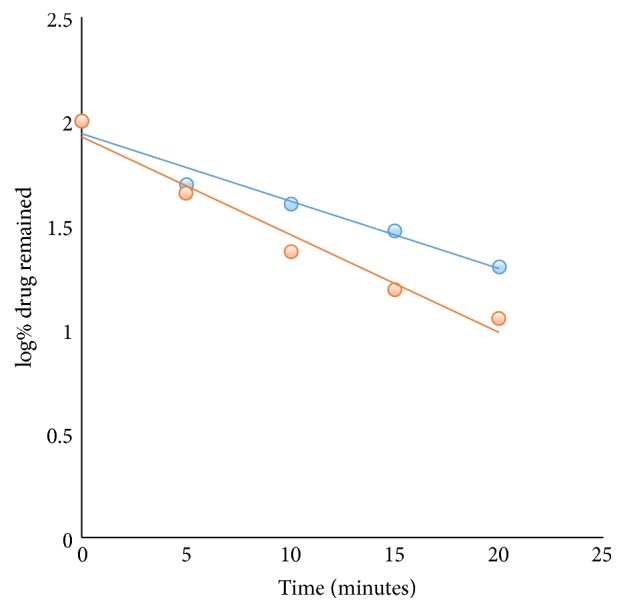
Effect of temperature (40°C, 50°C) on the degradation of DD in acidic conditions.

**Figure 7:**
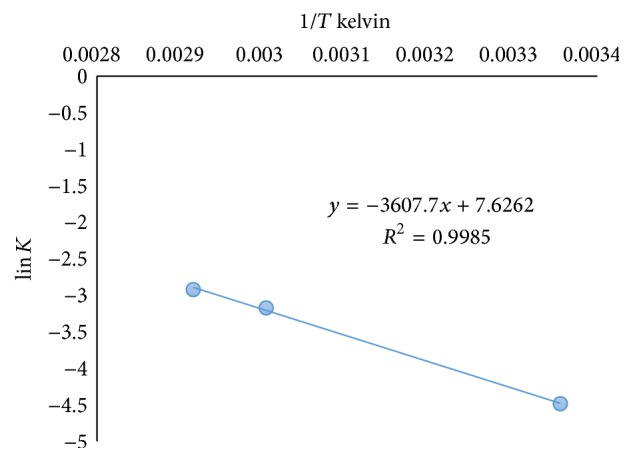
Arrhenius plot for degradation of DD at 40°C and 50°C.

The degradation process was monitored at different temperatures (40°C and 50°C) using the developed method. The linear plot of log% remained versus time interval (Figure 6) indicated the dependence of degradation rate and consequently *t*_1/2_ and *t*_90_ on temperature (Table 3). Arrhenius plot (Figure 7) was used to calculate the activation energy (7.48 kcal/mole) which was then utilized to calculate *t*_1/2_ and *t*_90_ at different temperatures.

The developed method was also applied to study the effect of alkali, hydrogen peroxide, and light on degradation of DD. No degradation products were observed under study conditions. Nevertheless, remarkable decrease in DD quantity (66.67%) was observed upon exposure to direct sunlight. Thus, protection from direct light is highly recommended for reconstituted samples. Decrease in DD quantity was also observed within two days; however, addition of PHZ stabilized DD wet sample for two weeks.

### 3.4. Stability Studies on PHZ

Contrary to DD, PHZ was proved to be stable under alkaline, acidic, oxidative, and photolytic conditions even at high temperature.

## 4. Conclusions

An accurate, precise, and feasible first derivative spectrophotometric method was developed for simultaneous determination of DD and PHZ in bulk and pharmaceutical dosage forms. The method was proved to be stability indicating and successfully resolved DD in the presence of its degradation products. The degradation of DD under acidic condition and its photo-instability were confirmed while PHZ was found to be stable under all the study conditions. Thus, the developed method could be successfully used for stability studies and routine quality control analysis of both drugs.

## Figures and Tables

**Table 1:** Linearity data of the developed method.

Parameter	DD	PHZ
Slope ± SD	0.0014 ± 0.00004	0.0018 ± 0.00003
Intercept ± SD	−0.0006 ± 0.0003	−0.0006 ± 0.0003
Correlation coefficient *r*^2^	0.9973	0.9990
Residual sum of squares	2.04 × 10^−7^	2.08 × 10^−7^
95% confidence interval of the slope	0.001229–0.001489	0.0017–0.0019
95% confidence interval of the intercept	−0.00149–0.00025	−0.00148–0.00027
LOD	0.63 *μ*g/mL	0.48 *μ*g/mL
LOQ	1.92 *μ*g/mL	1.47 *μ*g/mL

**Table 2:** Within-day and between-day precision data for DD and PHZ.

Drugs	Concentration (*μ*g/mL)	Within-day RSD%	Between-day RSD%
DD	6	2.13	0.81
	8	1.74	0.79
	10	0.51	2.68

PHZ	7.49	1.46	0.40
	9.98	1.43	0.44
	12.48	0.39	0.98

**Table 3:** Effect of temperature on DD degradation rate.

Temp.	Rate constant (min^−1^)	*t*_1/2_ (min.)	*t*_90_ (min.)
25°C	0.024	28.88	4.38
40°C	0.075	9.24	1.04
50°C	0.108	6.42	0.97
